# Impact of five different medial patellofemoral ligament-reconstruction strategies and three different graft pre-tensioning states on the mean patellofemoral contact pressure: a biomechanical study on human cadaver knees

**DOI:** 10.1186/s40634-018-0140-x

**Published:** 2018-06-28

**Authors:** Daniel Dornacher, Sabine Lippacher, Manfred Nelitz, Heiko Reichel, Anita Ignatius, Lutz Dürselen, Andreas Martin Seitz

**Affiliations:** 1 Department of Orthopaedics, Ulm University Medical Centre, Oberer Eselsberg 45, 89081 Ulm, Germany; 2 MVZ Oberstdorf, Clinics Kempten-Oberallgäu, Trettachstr. 16, 87561 Oberstdorf, Germany; 3 Institute of Orthopaedic Research and Biomechanics, Ulm University Medical Centre, Helmholtzstr. 14, 89081 Ulm, Germany

**Keywords:** Medial patellofemoral ligament, MPFL, Reconstruction, Patellofemoral contact pressure, Patellofemoral instability, Graft tensioning

## Abstract

**Background:**

The medial patellofemoral ligament (MPFL) is the main stabiliser of the patella and thus mostly reconstructed in the surgical treatment of patellofemoral dislocation. The aims of this study were to gain a better understanding of the influence of altered MPFL graft-fixation locations and different graft pre-tensions on patellofemoral contact pressure.

**Methods:**

Six human cadaveric knee joints were placed in a six-degree-of-freedom knee simulator. Mean PFCP (mPFCP) was evaluated in knee flexion of 0, 30 and 90° using a calibrated pressure-measurement system. After data assessment of the native knee joint, five MPFL reconstruction conditions were conducted: Anatomical double bundle; non-anatomical proximal patellar; non-anatomical distal patellar; non-anatomical proximal femoral; non-anatomical ventral femoral. The gracilis graft was fixed at a defined knee flexion of 30° and pre-tensioned to 2, 10 and 20 N.

**Results:**

Kruskal-Wallis testing resulted in no mPFCP differences between the native and anatomical reconstruction states.

Comparing the native and anatomical reconstruction states with the non-anatomical reconstruction states, no difference in the mPFCP both in knee extension (0°) (*p*>0.366) and in 30° knee flexion (*p*>0.349) was found. At 90° knee flexion, the following differences were identified: compared to the native knee state, the mPFCP increased after non-anatomical proximal femoral and non-anatomical ventral femoral reconstruction by 257% (*p*=0.04) and 292% (*p*=0.016), respectively. Compared to the anatomical reconstruction state, the mPFCP increased after non-anatomical proximal femoral reconstruction by 199% (*p*=0.042).

**Discussion and Conclusions:**

With respect to all study findings and to restore a physiological PFCP, we recommend using the anatomical footprints for MPFL reconstruction and a moderate graft pretensioning of 2-10 N.

## Background

With a prevalence of 5.8 in 100 k and an incidence of 23.2 up to 77.4 in 100 k, primary patellofemoral dislocation is considered as one of the most frequent knee-joint disorders (Colvin and West 2008; Fithian et al. 2004; Sanders et al. 2018; Sillanpaa et al. 2008). To prevent further dislocations, cartilage damage or persistent anterior knee pain, surgical patella stabilisation is recommended (Deie et al. 2005). Untreated patellofemoral instability commonly results in retropatellar osteoarthritis (Hawkins et al. 1986; Parikh et al. 2013; Salonen et al. 2017; Sanders et al. 2017).

The reconstruction of the medial patellofemoral ligament (MPFL) has been generally accepted in the surgical treatment of patellofemoral instability. To determine the most effective treatment to restore the natural MPFL function, different surgical techniques have been described, including variations in the reattachment at different anatomical sites (Arendt 2010; Deie et al. 2005; Deie et al. 2003; Drez Jr. et al. 2001; Quirbach et al. 2012; Schottle et al. 2010).

The footprints of the MPFL have been characterised by several cadaveric examinations (Baldwin 2009; Desio et al. 1998; Feller et al. 1993; LaPrade et al. 2007; Nomura et al. 2002; Philippot et al. 2009; Smirk and Morris 2003; Tuxoe et al. 2002). It originates between the adductor tubercle and the medial epicondyle. In day-to-day clinical practice, the radiological insertion at the femoral site described by Schöttle et al. became widely accepted for MPFL reconstruction (Schottle et al. 2007). There are investigations on the effect of an incorrect femoral graft insertion, showing differing results in the non-anatomical MPFL reconstruction (Burrus et al. 2015; Nomura et al. 2000; Nomura and Inoue 2005; Smirk and Morris 2003; Steiner et al. 2006; Stephen et al. 2012). The MPFL inserts at the proximal two thirds of the medial patellar facet (Baldwin 2009; LaPrade et al. 2007; Nomura 1999; Nomura et al. 2002; Tuxoe et al. 2002). A recent biomechanical study hypothesised that one of the indicators for premature degeneration of the retropatellar articular cartilage is an increased patellofemoral contact pressure (PFCP) (Rood et al. 2015). However, other previous examinations focused rather on length-change patterns and isometry of the graft than on the PFCP after alteration of the patellar fixation (Steensen et al. 2004; Stephen et al. 2012).

In accordance with current literature (Colvin and West 2008; Deie et al. 2003; Philippot et al. 2012; Stephen et al. 2014) and to the best of our knowledge, the most reasonable angle at which the graft should be pre-tensioned is at 30° of knee flexion, representing the state where the patella becomes centred in the trochlea femoris. This assumption is also underlined by an in vivo tracking analysis, indicating a lateral shift of the patella at a knee flexion angle over 0–30°, while further knee flexion consequently moves the patella in the direction of the medial trochlea (Suzuki et al. 2012).

The impact of graft tensioning on the PFCP remains controversial. Biomechanical examinations demonstrated that a graft tensioning with low loads (2 N) was both sufficient to prevent an overcorrection (Beck et al. 2007) and able to restore the medial PFCP and the tracking of the patella similar to an intact joint (Steiner et al. 2006; Stephen et al. 2014). Another study showed that a maximum graft tensioning of 10 N was sufficient to restore the physiological tracking of the patella, whereas greater tensile forces of 20, 30 and 40 N increased the PFCP and resulted in an undesired overcorrection (Philippot et al. 2012).

In consideration of the recent literature this study was designed to give a comprehensive overview on the pitfalls of MPFL reconstruction surgery using identical environmental testing conditions.

The consequential aims of this study were to investigate the impact of different graft pre-tensions on the mean PFCP (mPFCP) and to gain a better understanding of the influence of altered patellar and femoral MPFL graft-fixation locations compared to the native, deficient and anatomically reconstructed states on the mPFCP. The following hypotheses were formulated:

(I) Increasing graft pre-tension leads to an increased mPFCP;
(II) In 30° knee flexion, where the MPFL graft is initially fixed and pre-tensioned, the mPFCP remains unaltered by the MPFL graft-fixation method;
(III) Anatomical double-bundle MPFL reconstruction restores the mPFCP pattern of the native knee;
(IV) Non-anatomical patellar and femoral graft fixations lead to an excessive mPFCP compared to the native state.

Experimental evidence in support of these hypotheses would suggest that non-anatomic reconstructions lead to a significantly higher mPFCP compared to the anatomic reconstructions.

## Methods

**Fig. 1:**
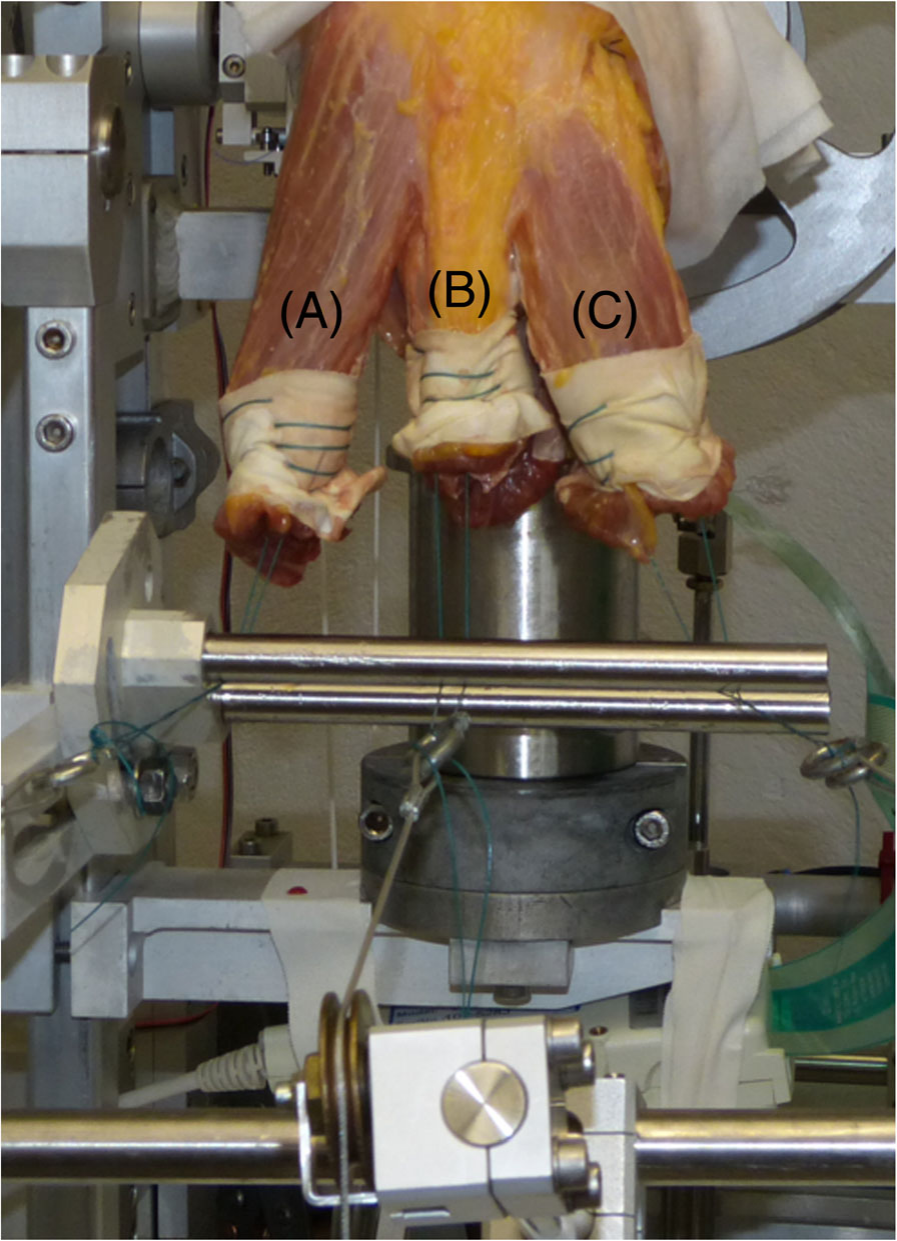
Cadaveric knee mounted in the knee-joint simulator. Simulation of the three main muscle groups according to Farahmand et al. (Farahmand et al. 1998) of the *M. quadriceps*: m. vastus lateralis [A: 75 N ≅ 42%], mm. rectus femoris and vastus intermedius [B: 60 N ≅ 35%] and m. vastus medialis [C: 40 N ≅ 23%], proportional to the physiological loading situation

The mPFCP was examined on six cadaver knee joints, comparing seven different MPFL conditions and three different graft pre-tensioning conditions during three knee motion cycles of 0–90° flexion using a previously described knee joint-motion and -loading simulator (Durselen et al. 2011) (Fig. 1).

### Specimen preparation

After IRB approval, eight human cadaver knee joints (ScienceCare, Phoenix, Arizona, USA) from adult body donors (mean 52.1 yrs., standard deviation 8.4 yrs) were used. Two of the eight specimens were used to optimize the test setup with special attention to the fixation of the pressure measurement system during pre-tests. Thus, resulting in a total of six knees for the biomechanical testing. Prior to preparation and in addition to reviewing patient history, radiographic examination (Siremobil Iso-C, Siemens, Germany) was performed to exclude any predisposition for patellofemoral instability or other relevant knee disorders.

Before testing, the specimens were thawed within 24 h. Subsequently, skin and subcutaneous tissue were removed, leaving the ligaments, required muscles, including tendons (mm. rectus femoris and vastus intermedius, m. vastus medialis, m. vastus lateralis), and joint capsule intact. The gracilis tendons were harvested and served as the MPFL graft. The fibula was resected to a total length of 6 cm and fixed to the proximal tibia using a tricortical positioning screw. The bony ends of the tibia and the femur were shortened to a length of 20 cm above and below the joint line and potted in polymethyl methacrylate (Technovit 3040, Heraeus Kulzer GmbH, Germany).

### Biomechanical testing

**Fig. 2:**
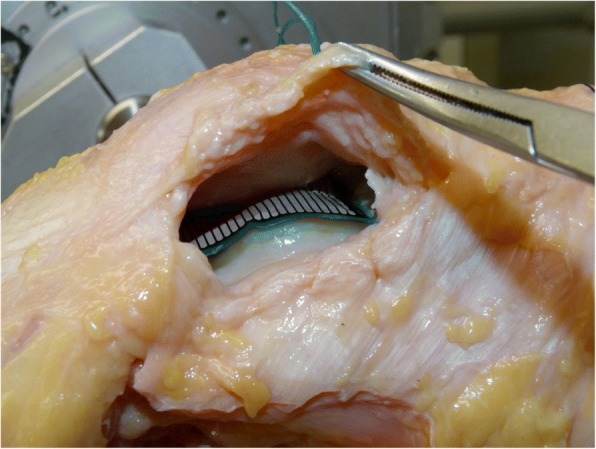
Position of the pressure-measurement sensor (Tekscan K-Scan) between the patella and the trochlea femoris

**Fig. 3:**
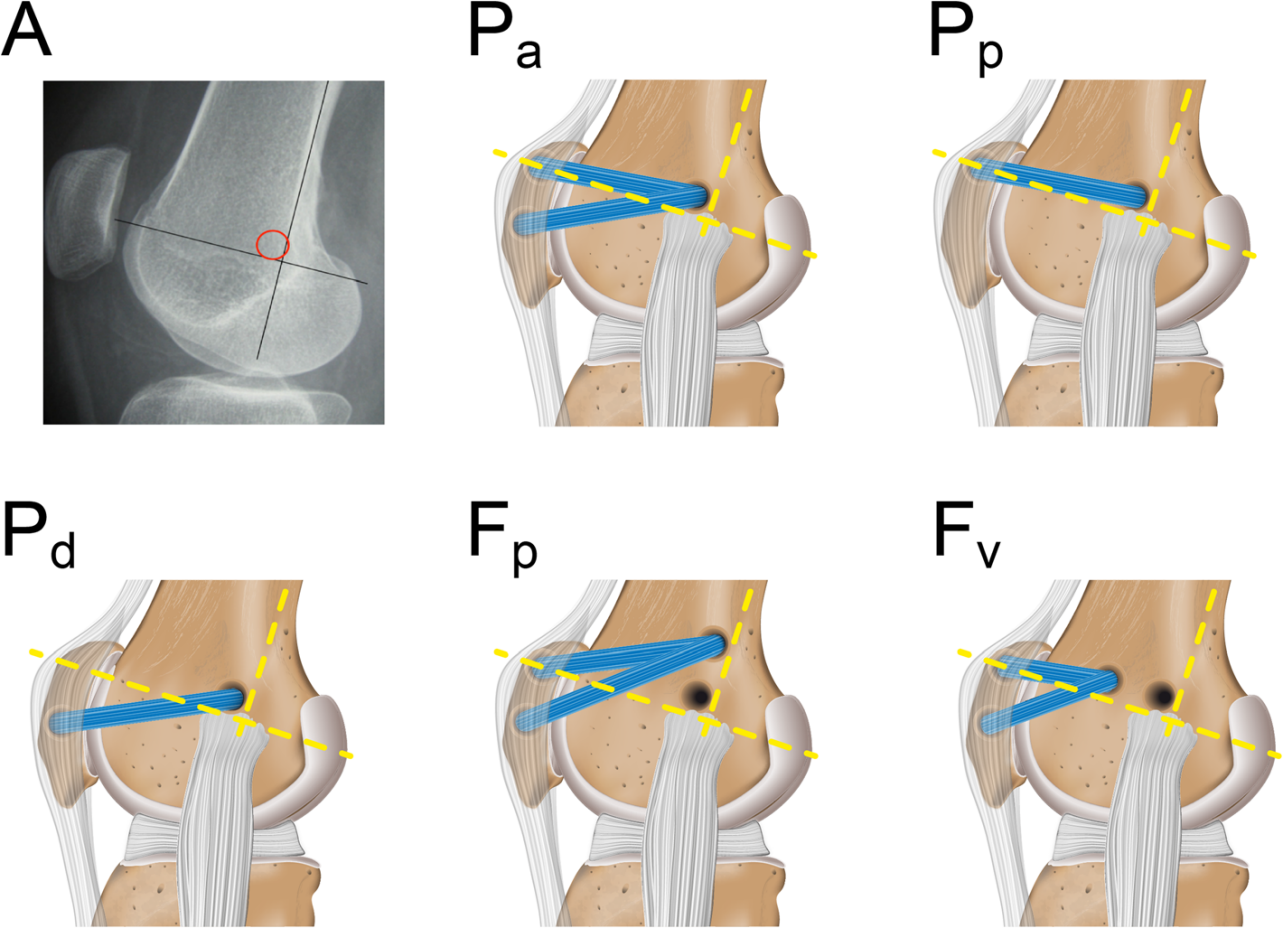
*A:* Representative X-ray indicating the Schoettle-Point (Schottle et al. 2007); P_a_: Anatomical double-bundle MPFL reconstruction using the anatomical, Schoettle-Point and both the proximal and distal patellar fixation points; P_p_: Non-anatomical single-bundle MPFL reconstruction using the anatomical, Schoettle-Point and the proximal patellar fixation point; P_d_: Non-anatomical single-bundle MPFL reconstruction using the anatomical, Schoettle-Point and the distal patellar fixation point; F_p_: Non-anatomical double-bundle MPFL reconstruction using a insertion 1 cm proximal to the Schoettle-Point and both the proximal and distal patellar fixation points; F_v_: Non-anatomical double-bundle MPFL reconstruction using a insertion 1 cm ventral to the Schoettle-Point and both the proximal and distal patellar fixation points. The yellow dotted lines indicate the reconstruction lines for the Schoettle-Point

Specimens were placed in a customised six-degree-of-freedom knee-joint simulator (Durselen et al. 2011), allowing continuous data acquisition of the knee motion during three flexion cycles of 0–90°. The quadriceps muscle was simulated using static weights that were attached to the individual heads of the quadriceps of a total of 175 N. In accordance with the literature (Farahmand et al. 1998; Stephen et al. 2014), the three relevant muscle groups of the quadriceps were separated to allow proportional simulation of the muscle forces (Fig. 1) (mm. rectus femoris and vastus intermedius [60 N ≅ 35%], m. vastus medialis [40 N ≅ 23%], m. vastus lateralis [75 N ≅ 42%]). The body weight was simulated using a static weight of 200 N following an established method (Durselen et al. 2011). The mPFCP was recorded during knee motion using a calibrated pressure-measurement system (K-Scan, Tekscan Inc., Boston, USA). Calibration of the sensor was conducted as previously described (Seitz et al. 2012). The sensor was centred at the trochlea femoris (Fig. 2) and fixed infrapatellar using a combination of tape and adequate sutures (Ethicon Vicryl 2–0, Johnson & Johnson Medical GmbH, Norderstedt, Germany). After data assessment of the native knee joint (P_nat_) and subsequent to dissection of the MPFL, the following five MPFL reconstruction techniques were performed (Fig. 3):

- Anatomical patellar double-bundle technique (P_a_)
- Non-anatomical, proximal patellar single-bundle technique (P_p_)
- Non-anatomical, distal patellar single-bundle technique (P_d_)
- Non-anatomical double-bundle fixation 1 cm proximal to the anatomical femoral insertion (F_p_)
- Non-anatomical double-bundle fixation 1 cm ventral to the anatomical femoral insertion (F_v_)

All anatomical and non-anatomical footprints for the insertions were individually identified using an image intensifier (Quirbach et al. 2012) (Fig. 3a). The free end of the gracilis graft was sutured to the lateral margin of the patella combined with a suture disk (Tendon Suture Disk, Merete Medical GmbH, Berlin, Germany). In contrast to interference and anchor screws, this fixation technique maintained an undamaged graft during all required tests of each respective knee. To identify the impact of graft tensioning on the mPFCP, the gracilis graft was fixed at a defined knee flexion of 30° and randomly pre-tensioned to 2, 10 and 20 N using static weights. Subsequently, three flexion-extension cycles were applied to the knees. With respect to the time-dependent behaviour of the soft tissues of the knee joint, only the third loading cycle was used for further data analysis. Although continuous data acquisition was achieved throughout knee flexion cycles of 0–90°, only data of 0, 30 and 90° knee flexion were further statistically analysed. Flexion of 30° was assumed to be the state where the patella centres in the trochlea femoris (Suzuki et al. 2012). The graft was pre-tensioned in this position. Furthermore, knee extension (0°) and 90° flexion were analysed, because these were assumed to be the states with the least and greatest contact pressure, respectively.

### Statistical analysis

Given a sample size of *n* = 6, post-hoc power analysis (G*Power 3.1.9.2. (Faul et al. 2009)) using a Wilcoxon signed-rank test (matched pairs: e.g. native MPFL versus proximal femoral insertion of the MPFL) revealed a power of 0.99 (1-β error probability, effect size 3.15, α=0.05). Because of the low sample size, data were assumed not to be normally distributed. Further statistical analyses were performed using a statistical software package (SPSS Ver. 20, IBM Inc., USA). A value of *p*≤0.05 was considered to be significant.

(I) Effect of graft pre-tensioning of each reconstruction state was independently evaluated in 0, 30 and 90° knee flexion using a Wilcoxon signed-rank test for matched pairs.
(II) mPFCP of all knee states (P_nat_, P_a_, P_p_, P_d_, F_v_, F_p_) at the knee flexion (30°), where the MPFL graft was initially fixed and pre-tensioned, was investigated using a Kruskal-Wallis test followed by a post-hoc Bonferroni-Dunn test when significant differences were detected.
(III) mPFCP of the P_nat_ was compared to P_a_ in 0, 30 and 90° knee flexion using a Kruskal-Wallis test followed by a post-hoc Bonferroni-Dunn test when significant differences were detected.
(IV) mPFCP of the P_nat_ and the P_a_ were compared to the non-anatomical reconstruction states (P_p_, P_d_, F_p_, F_v_) in 0, 30 and 90° knee flexion using a Kruskal-Wallis test followed by a post-hoc Bonferroni-Dunn test when significant differences were detected.

## Results

### Graft pre-tensioning

**Fig. 4:**
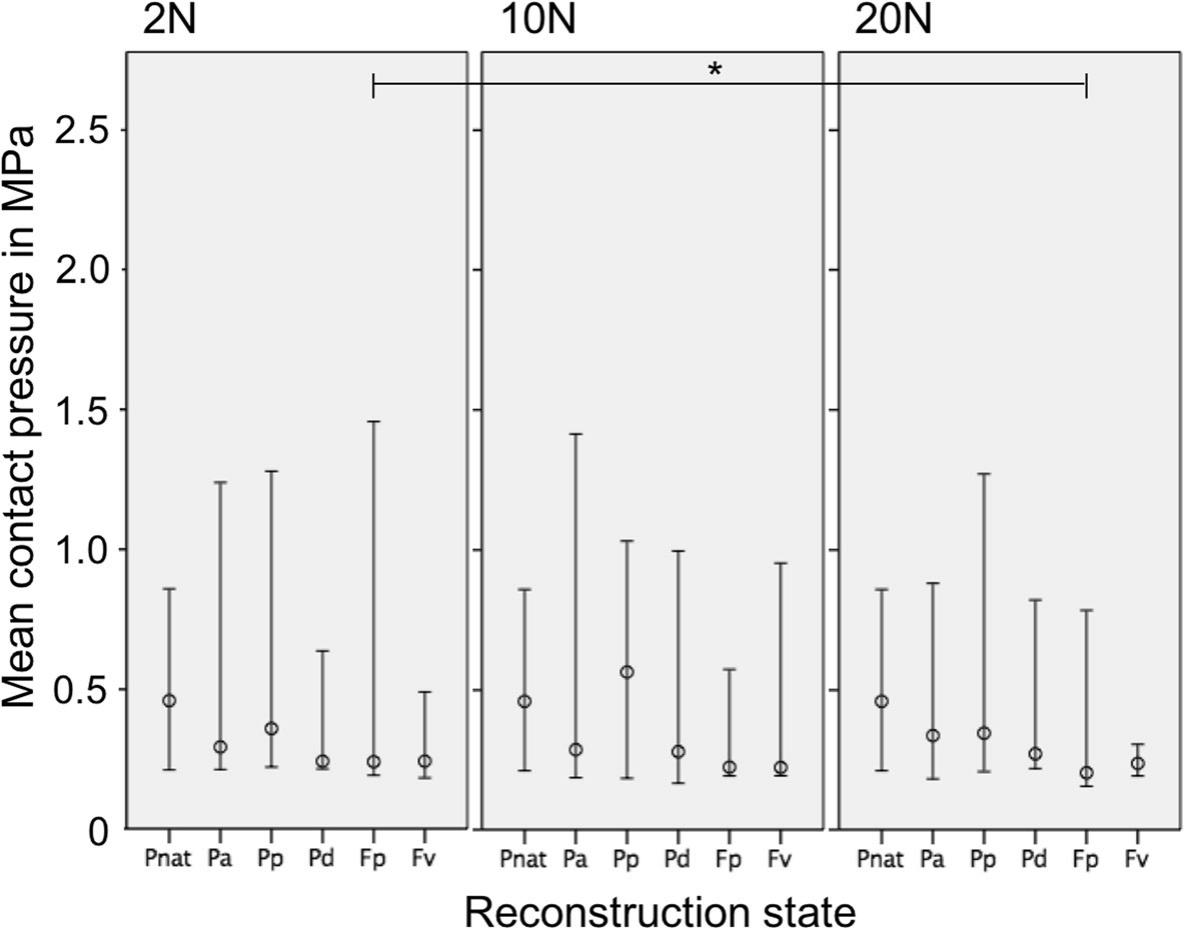
Mean patellofemoral contact pressure (median, minimum, maximum) at 0° knee extension and with 2, 10 and 20 N graft pre-tensioning (P_nat_: Native knee joint; P_a_: Anatomical patellar double-bundle technique; P_p_: Non-anatomical, proximal patellar single-bundle technique; P_d_: Non-anatomical, distal patellar single-bundle technique; F_p_: Non-anatomical double-bundle fixation 1 cm proximal to the anatomical femoral insertion; F_v_: Non-anatomical double-bundle fixation 1 cm ventral to the anatomical femoral insertion). *p≤0.05

**Fig. 5:**
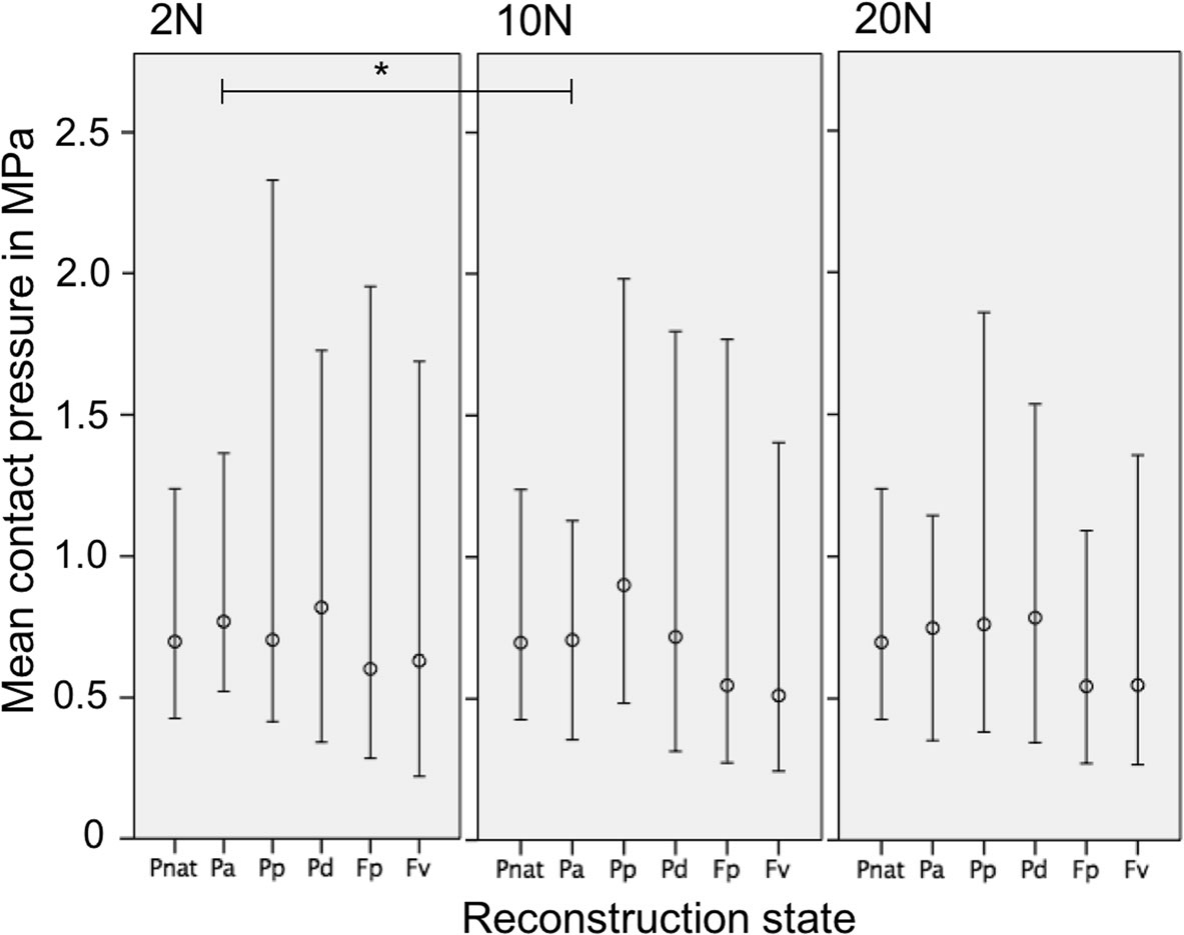
Mean patellofemoral contact pressure (median, minimum, maximum) at 30° knee flexion and with 2, 10 and 20 N graft pre-tensioning (P_nat_: Native knee joint; P_a_: Anatomical patellar double-bundle technique; P_p_: Non-anatomical, proximal patellar single-bundle technique; P_d_: Non-anatomical, distal patellar single-bundle technique; F_p_: Non-anatomical double-bundle fixation 1 cm proximal to the anatomical femoral insertion; F_v_: Non-anatomical double-bundle fixation 1 cm ventral to the anatomical femoral insertion). *p≤0.05

**Fig. 6:**
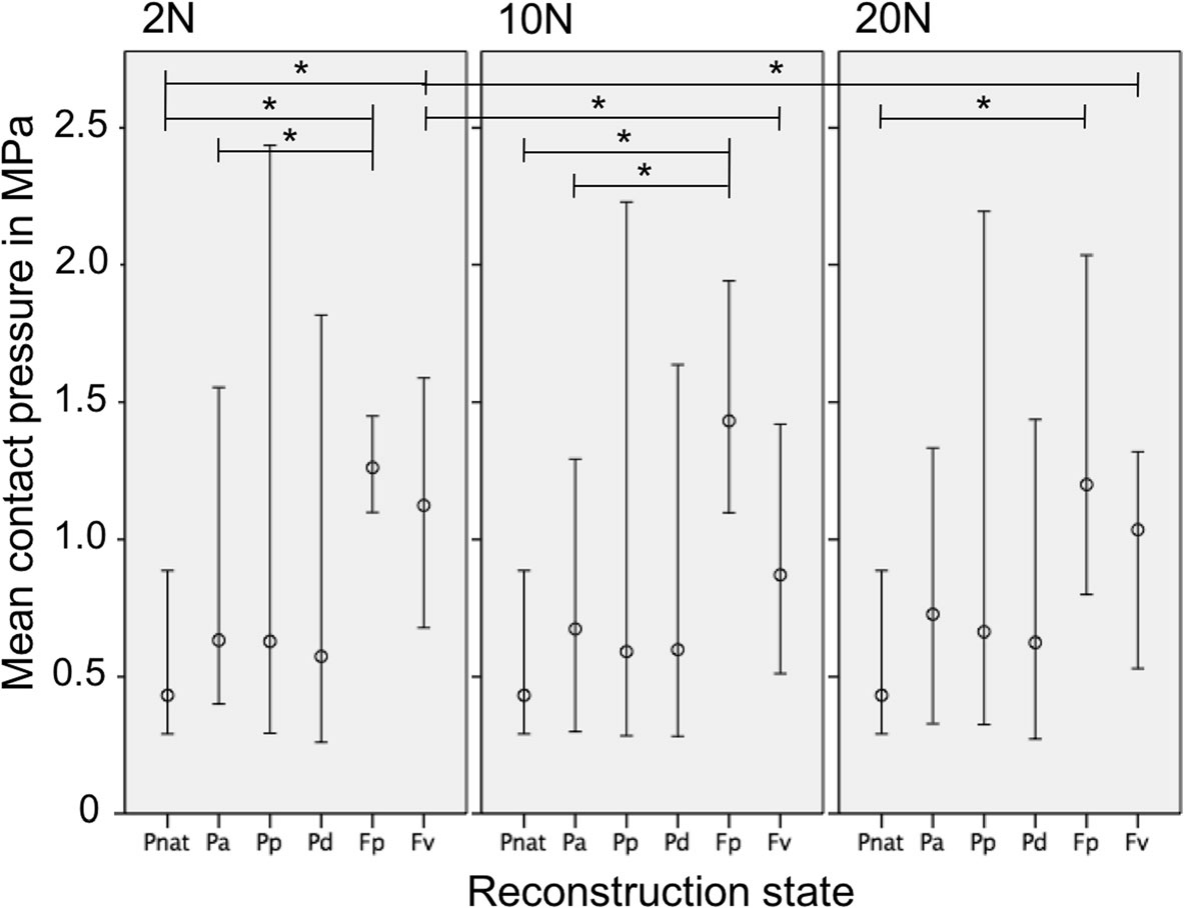
Mean patellofemoral contact pressure (median, minimum, maximum) at 90° knee flexion and with 2, 10 and 20 N graft pre-tensioning (P_nat_: Native knee joint; P_a_: Anatomical patellar double-bundle technique; P_p_: Non-anatomical, proximal patellar single-bundle technique; P_d_: Non-anatomical, distal patellar single-bundle technique; F_p_: Non-anatomical double-bundle fixation 1 cm proximal to the anatomical femoral insertion; F_v_: Non-anatomical double-bundle fixation 1 cm ventral to the anatomical femoral insertion). *p≤0.05

When increasing the pre-tensioning from 2 to 20 N at full knee extension (0°) and after non-anatomical proximal femoral reconstruction (F_p_) the median mPFCP significantly decreased (*p* = 0.04) from 0.24 to 0.20 MPa (Fig. 4). The same pattern was detected at 30° knee flexion and after anatomical reconstruction (P_a_), where the mPFCP significantly decreased (p = 0.04) under a pre-tensioning of 10 N (0.71 MPa) compared to 2 N (0.77 MPa) pre-tensioning (Fig. 5). In 90° knee flexion, a significantly different mPFCP was observed between 2 and 10 N (*p* = 0.04) and between 2 and 20 N (*p* = 0.046) of graft pre-tensioning after non-anatomical ventral femoral reconstruction (F_v_). In these cases, the median mPFCP decreased under a higher pre-tension from 1.12 to 0.87 MPa and from 1.12 to 1.04 MPa, respectively (Fig. 6).

*Initial knee flexion state (30°) where the graft was fixed and pre-tensioned*

Comparing the different reconstruction states, no differences for the mPFCP were observed (*p* > 0.349) (Fig. 5). Consequently, further statistical analyses were performed without the 30° knee-flexion states.

### Patellofemoral contact pressure of P_nat_ compared to P_a_

Kruskal-Wallis testing produced no mPFCP differences between the native (P_nat_) and anatomical reconstruction (P_a_) states for all graft pre-tensioning states and under 0° (*p* > 0.987) and 90° (*p* > 0.577) knee flexion.

### Patellofemoral contact pressure of P_nat_ and P_a_ compared to P_p_, P_d_, F_p_ and F_v_

When comparing the native and the non-anatomical reconstruction states, the mPFCP was not different in knee extension (0°) for all graft pre-tensioning states (*p* > 0.366) (Fig. 4). At a knee flexion of 90°, the following differences were observed: with a graft pre-tensioning of 2 N, the mPFCP of the non-anatomical proximal femoral reconstructed knee (F_p_) compared to the native knee (P_nat_) was increased by 257% (*p* = 0.04) and the ventral femoral reconstructed (F_v_) knee versus the native knee increased by 292% (*P* = 0.016). Furthermore, with a graft pre-tensioning of 10 N and 20 N, the non-anatomical proximal femoral reconstructed knees (F_p_) were significantly increased by 331% (*p* = 0.001) and 278% (*p* = 0.004), respectively, compared to the native-knee state (Fig. 6).

In 0° knee flexion, the mPFCP displayed no differences between the state of anatomical MPFL reconstruction (P_a_) and the states of non-anatomical reconstruction with a graft pre-tensioning of 2, 10 or 20 N (*p* > 0.756). At a knee flexion of 90°, differences for the following conditions were observed: comparing anatomical reconstruction (P_a_) to non-anatomical proximal femoral reconstruction (F_p_), with a graft pre-tensioning of 2 N the mPFCP increased by 199% (*p* = 0.042) and with a graft pre-tensioning of 10 N by 213% (*p* = 0.006) (Fig. 6).

## Discussion

The present study design was appropriate to respond to the proposed hypotheses: Hypothesis (I) was disproved, because increased graft pre-tension did not lead to an increased mPFCP. The hypotheses (II) and (III) were proved: In 30° knee flexion, where the graft was fixed and pre-tensioned, the location of the graft fixation and its pre-tension did not alter the mPFCP. The mPFCP pattern of the native knee was restored by the anatomical double-bundle MPFL reconstruction. Hypothesis (IV) was proved for the non-anatomical femoral fixation but disproved for the non-anatomical patellar fixation: The non-anatomical femoral fixations led to an excessive mPFCP compared to the native state and the anatomical reconstruction, whereas the non-anatomical patellar fixations did not display this effect.

With regard to the above-mentioned hypotheses, there were four main findings in this biomechanical study: First, an increase of graft pre-tensioning from 2 to 10 N and finally to 20 N did not lead to an increased mPFCP. Second, when the graft was fixed in 30° knee flexion, the method of fixation did not alter the mPFCP. Third, anatomical double-bundle MPFL reconstruction was able to almost restore the mPFCP compared to the native knee. Fourth, at a knee flexion of 90°, a non-anatomical ventral or proximal positioning of the graft at the femur led to an excessive mPFCP. By contrast, a non-anatomical positioning of the graft at the patella, either at the proximal or distal margins, had no relevant impact on the mPFCP.

### Graft pre-tensioning

It is imperative during MPFL reconstruction to avoid graft over-tensioning (Thaunat and Erasmus 2009). The present study demonstrated that a graft pre-tensioning ranging from 2 to 20 N does not increase the mPFCP. In contrast to our expectations, the mPFCP slightly decreased when increasing the pre-tension. This could be explained by altered patellar kinematics after non-anatomical MPFL reconstruction: In 0° knee extension, a non-anatomical rather proximal femoral graft fixation might lead to a proximalisation of the patella under higher preloads, resulting in a larger contact area and thus, in a lower mean pressure. The same might be true at 90° knee flexion. A reconstruction where the femoral insertion is 1 cm ventralised might result in an according ventralisation of the patella. In accordance with our findings, Beck et al. found that a 2 N graft tension restored the native translation of the patella (Beck et al. 2007). Compared to the present study, the authors used both a comparable test method and simulation of muscle forces. In their study, the application of 2 and 10 N to the graft had no effect on the medial mPFCP. However, they found a significant increase of the medial mPFCP when the graft was pre-tensioned to a 40 N load. By contrast, we used 20 N as the maximum pre-tensioning force. Therefore, it appears possible that a graft pre-tensioning of 20 N is still within the physiological range without significantly impacting the PFCP. Philippot et al. demonstrated in a biomechanical study on six cadaveric knees that 10 N pre-tensioning are sufficient to restore a normal patellar tilt, lateral shift and rotation (Philippot et al. 2012). Furthermore, the authors demonstrated that a graft tensioning of 20, 30 and 40 N caused an increase of the PFCP and thus, an undesired overcorrection. The difference, compared to our findings, could be explained by the comparatively low quadriceps loads of 10 N, which may have led to a more instable patella in their study. This instability may then lead to a greater contact pressure on the medial facet of the femoral groove and thus to a higher local PFCP. In a recent study, it was demonstrated that a graft tension of 2 N restored physiological patellar tracking (Stephen et al. 2014). Supporting this and also reflecting our findings, in the day-to-day clinical practice, a graft pre-tensioning of 2–10 N appears reasonable. To the best of our knowledge, there is only one available study describing the influence of the knee flexion angle on the fixation stability of the MPFL reconstruction (Stephen et al. 2016). In their investigation, Stephen et al. varied the knee flexion angle between 0° and 60° and measured the PFCP consecutively. Finally, they concluded, that when starting at a knee flexion angle of 30°, the patella enters the trochlea and centres itself. Therefore, the authors recommend a fixation of the graft at a knee flexion angle between 30° and 60°. This is in accordance with the findings of the present study.

### Patellofemoral contact pressure of P_nat_ compared to P_a_

Comparing the mPFCP of the native (P_nat_) and the anatomically reconstructed (P_a_) MPFL knees resulted in no difference in full knee extension or in 90° knee flexion. The similar behaviour for the mPFCP after anatomical double-bundle reconstruction of the MPFL using a gracilis graft compared to the native state underlines the coherence of the landmarks in reconstruction surgery to the anatomical footprints of the MPFL (Baldwin 2009; Feller et al. 1993; LaPrade et al. 2007; Nomura et al. 2002; Philippot et al. 2009; Smirk and Morris 2003; Tuxoe et al. 2002). The findings in the present study confirmed those of Stephen et al. (Stephen et al. 2014). In their biomechanical study, the authors demonstrated that an alteration of the femoral footprint in MPFL reconstruction surgery resulted in a significant increase of the mean and peak PFCP. In accordance with our findings, Stephen et al. stated that a correct femoral tunnel positioning restored joint kinematics and the PFCP to physiological conditions.

### Patellofemoral contact pressure of P_nat_ and P_a_ compared to P_p_, P_d_, F_p_ and F_v_

The comparison of the mPFCP after anatomical reconstruction of MPFL (P_a_) with non-anatomical graft fixations (F_v_, F_p_, P_d_, P_p_) displayed an analogous pattern to the comparison of the mPFCP of the native knee with the above-mentioned non-anatomical graft fixations, given a graft pre-tension of 2, 10 and 20 N. This leads to the assumption that an anatomically reconstructed MPFL leads to a similar biomechanical outcome in terms of the mPFCP as the native knee. Similar findings were observed by Stephen et al. (Stephen et al. 2016), who used different MPFL graft types (double-bundle gracilis, quadriceps tendon, tensor fasciae latae allograft). Regardless of the grafts and in agreement with our results, the PFCP was only altered by the femoral tunnel placement. Other authors reported the most distinct difference in the PFCP between the native knee and the non-anatomically reconstructed MPFL knee at high knee-flexion angles (Stephen et al. 2014). This might result from the extended force that is applied to the retropatellar surface by an increasingly stretched quadriceps during knee flexion. This correlates with our findings, where a non-anatomical femoral positioning of the graft 1 cm proximal (F_p_) or ventral (F_v_) resulted in an increased PFCP at 90° knee flexion of 331% compared to the native state. In computational models, Elias and Cosgarea demonstrated that a proximal malposition of the femoral attachment combined with a short graft leads to an extensive increase in the medial PFCP (Elias and Cosgarea 2006). Furthermore, Stephen et al. reported a strong relationship between the patellofemoral contact and the positioning of the graft in the MPFL reconstruction (Stephen et al. 2014). They found a significant increase in the peak medial PFCP during knee flexion and extension with a femoral graft positioned just 5 mm proximal or distal to its anatomical insertion. In accordance with our findings, they reported that an anatomically positioned graft was able to restore the joint pressure and the patellar tracking. Stephen et al. concluded, from a biomechanical point of view, that the correct placement of the femoral insertion is crucial for the outcome of an MPFL reconstruction. Concomitant with these findings, Camp et al. stated in their clinical study that a non-anatomical MPFL repair at the femoral condyle appears to be the only relevant risk factor in MPFL reconstruction surgery (Camp et al. 2010). This is further underlined by the work of Bollier et al., who performed an analysis of failure in MPFL reconstruction and reported a high correlation between femoral graft malposition and graft failure (Bollier et al. 2011). In previous biomechanical examinations, different patellar insertions of the MPFL were analysed, focusing on graft isometry and length-change patterns. Steensen et al. observed no significant differences for the graft isometry using three patellar attachments (Steensen et al. 2004). Stephen et al. demonstrated a significant influence of the graft length (Stephen et al. 2012). Although the PFCP was not determined in their study, the negative impact of non-anatomical patellar graft fixation in biomechanical investigations was underscored.

### Limitations

Like all controlled laboratory studies, the present biomechanical investigation also has limitations. The *M. quadriceps* was simulated using a constant total load of 175 N. Physiological-like quadriceps muscle simulation results in a higher peak PFCP (Goudakos et al. 2009) compared to the peak PFCP of the present study (results not shown). Therefore, we assume, that the mPFCP would also be higher with physiological-like muscle-force simulation. However, because the loading patterns of the PFCP mainly depend on the MPFL reconstruction technique, the respective differences in the mPFCP would be the same. The experiments were performed using specimens without a history of patellofemoral instability. It has been described that trochlear dysplasia is one of the most common predispositions for patellofemoral instability (Colvin and West 2008). Within this disease, the patella is not centred physiologically in a dysplastic trochlea, which might lead to a different patellofemoral contact pattern. However, to what extent the mPFCP would be changed is difficult to say. Therefore, we would recommend to investigate the effects by using pathological specimens in a future study. Lastly, the pressure-measuring system was placed on the cartilage surface of the trochlea femoris and fixed sutures. Consequently, the friction between the interacting foil and cartilage surfaces could increase, which might have influenced the kinematics of the patellofemoral joint. Nevertheless, there is no clear evidence in literature that the friction might have had an influence on the measurements. Additionally, a study investigating the accuracy and repeatability of the Tekscan sensor in patellofemoral contact measurements (Wilson et al. 2003) reported a positive effect of the altered friction, finally resulting in a better entrapment of the sensor during continuous movements. In general, the Tekscan sensors are recommended in measuring the patellofemoral contact mechanics (Wilharm et al. 2013; Wilson et al. 2003).

## Conclusions

To restore a physiological mPFCP in MPFL reconstruction surgery, this biomechanical study emphasises that particularly in the femoral graft placement, care should be taken to insert the graft in the most anatomical way. Particularly in a knee flexion of 90°, the mPFCP was dramatically increased when the graft was positioned wrongly at the femoral side. Furthermore, we found only a minor impact on the mPFCP when the graft was solely placed non-anatomically at the distal or the proximal margin of the patella. Surprisingly, we found no significantly increasing effect on the mPFCP when elevating the graft pre-tensioning force. This leads to the conclusion that with regard to the current literature and to the feasibility in the day-to-day clinical practice, a graft pre-tensioning with a range of 2–10 N appears reasonable. From a biomechanical point of view, the present study implies that the anatomical MPFL graft placement is considered to be the most preferred reconstruction method for primary patellofemoral dislocation or other patellar injuries that might lead to retropatellar osteoarthritis. For a better understanding of the PFCP in knees with a dysplastic trochlea, we recommend to investigate both the PFCP changes in pathological knees and the influence of different muscle-force simulations in future biomechanical studies.

## Abbreviations

F_p_: Non-anatomical double-bundle MPFL reconstruction state, 1 cm proximal to the anatomical femoral insertion
F_v_: Non-anatomical double-bundle MPFL reconstruction state, 1 cm ventral to the anatomical femoral insertion
mPFCP: Mean patellofemoral contact pressure
MPFL: Medial patellofemoral ligament
P_a_: Anatomical patellar double-bundle MPFL reconstruction state
P_d_: Non-anatomical, distal patellar single-bundle MPFL reconstruction state
PFCP: Patellofemoral contact pressure
P_nat_: Native knee state
P_p_: Non-anatomical, proximal patellar single-bundle MPFL reconstruction state
